# The Effect of the *G*_1_ - S transition Checkpoint on an Age Structured Cell Cycle Model

**DOI:** 10.1371/journal.pone.0083477

**Published:** 2014-01-09

**Authors:** Gary S. Chaffey, David J. B. Lloyd, Anne C. Skeldon, Norman F. Kirkby

**Affiliations:** 1 Department of Mathematics, University of Surrey, Surrey, England; 2 Department of Chemical Engineering, University of Surrey, Surrey, England; University of Torino, Italy

## Abstract

Knowledge of how a population of cancerous cells progress through the cell cycle is vital if the population is to be treated effectively, as treatment outcome is dependent on the phase distributions of the population. Estimates on the phase distribution may be obtained experimentally however the errors present in these estimates may effect treatment efficacy and planning. If mathematical models are to be used to make accurate, quantitative predictions concerning treatments, whose efficacy is phase dependent, knowledge of the phase distribution is crucial. In this paper it is shown that two different transition rates at the 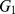-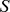 checkpoint provide a good fit to a growth curve obtained experimentally. However, the different transition functions predict a different phase distribution for the population, but both lying within the bounds of experimental error. Since treatment outcome is effected by the phase distribution of the population this difference may be critical in treatment planning. Using an age-structured population balance approach the cell cycle is modelled with particular emphasis on the 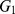-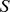 checkpoint. By considering the probability of cells transitioning at the 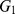-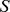 checkpoint, different transition functions are obtained. A suitable finite difference scheme for the numerical simulation of the model is derived and shown to be stable. The model is then fitted using the different probability transition functions to experimental data and the effects of the different probability transition functions on the model's results are discussed.

## Introduction

The cell cycle is an ordered set of events that a cell undergoes from its birth until it divides into two daughter cells [1]. In eukaryotic cells the cell cycle may be broken down into four distinct phases, namely 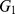, 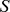, 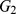 and 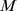. After birth, a cell enters the longest of the phases, the 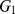 (Gap 1) phase, during which the cell takes on nutrients needed to complete the rest of the cycle. Once the cell has absorbed enough nutrients it may proceed round the cell cycle leaving the 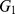 phase and entering the 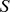 (Synthesis) phase. Not all cells leave the 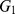 phase to enter the 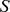 phase, a number of cells enter a quiescent period where they remain viable but leave the cell cycle for a short time, these cells enter the 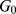 (Gap 0) phase. During the 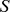 phase a cell replicates its DNA, at the end of which they have effectively doubled their DNA content. Once DNA synthesis is completed the cell enters the 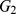 (Gap 2) phase. During the 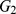 a cell grows in size and prepares for mitosis. Upon leaving 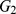 the final phase 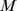 (Mitosis) is entered. It is during the mitotic phase that the cell divides, producing two daughter cells. Due to the processes involved in cell division, cells in the 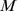 phase are especially vulnerable to radiotherapy. It should be noted that the 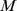 phase may be broken down further into several sub phases, however this is of no consequence for the model discussed herein. The actual length of the cell cycle is variable, this variability mainly occurs in the length of time cells spend in the 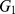 phase which is governed by the way in which cells ‘transition’ from the 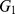 phase to the 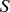 phase [2]. Once a cell commits itself to DNA synthesis (i.e. enters the 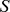 phase) it must continue the cell cycle until division is complete, the ‘transition’ from the 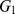 phase to the 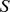 phase is irreversible.

Chemotherapy drugs can be divided into several types, each of which target a specific process within the cell cycle such as RNA synthesis or cell division. Hence the efficacy of many chemotherapy drugs (e.g. [3], [4] and [5]) is dependent on the cell cycle phase. The radiosensitivity of cells is also phase dependent (e.g. [6], [7] and [8]) with cells in the 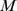 (mitotic) phase having their chromosomes arranged in a line prior to separation making them particularly sensitive to ionising radiation. Due to the phase dependent nature of chemotherapy drugs and radiotherapy knowledge of how the cells progress through the different phases is crucial.

There have been a number of mathematical models developed for populations of cells progressing round the cell cycle. Systems of ordinary differential equations may be used to model the growth kinetics of populations of cells however these are too simplistic to capture the intrinsic properties of the cell cycle, but are often an invaluable first step in understanding the kinetics of a population of cells. To adequately model crucial properties of a population of cells such as age, mass or DNA distribution a system of partial differential equations is needed.

Many partial differential equation models share the same fundamental population balance structure as detailed in [9], [10] and [11]. These models may broadly be grouped in terms of which property of the cell is used to structure the model, the main properties used being DNA ([12], [13], [14], [15] and [16]), age ([17], [18], [19] and [20]) and mass ([17], [21], [19]).

There are advantages of using a DNA or mass structured model in as much that these quantities may be easily determined experimentally, however such a model contains no information about the age of a particular cell and as such it is possible for cells to remain in the cycle for an infinite amount of time. By use of an age-structured model it is possible to control the length of time a cell may remain in the cell cycle, in particular the 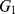 phase. Another advantage of age structuring is that, if growth rates and nutrient uptake rates for a given cell line are known, it is possible to determine the mass and DNA content of a cell from its age, however given the cells DNA content or mass it is not possible to determine a cell's age as there is not a one-to-one mapping between age and DNA or mass.

Analysis has been undertaken to determine the existence and stability of steady size/DNA distributions [22] which may occur under specific circumstances using an age structured model. Population balance models have been used not only on healthy, unperturbed cell lines but also to model the effects of various treatments to cancer cell populations [15], [18], [16] and [23].

In this paper, an age structured cell cycle model is considered together with two different functions governing the movement between the 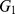 and 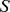 phases. Whilst, different functions have been used in the past [18], [20] and [24] little has been done to study the effects of different functions on the phase distributions of cells. It is shown that it is possible to obtain very similar growth curves using different transition functions with the fundamental difference being in the phase distributions for the cells. Although the differences in the phase distributions lie within the range of experimental error for many techniques such as conventional flow cytometry it may be significant in terms of treatment optimisation. The purpose of this paper is to understand how different transition rules may effect the phase distribution of the cells and that whilst the motivation for this analysis is the phase dependent nature of certain treatments these have not been included within the model.

This paper is outlined as follows. The age structured model is presented in Section 1 together with a brief overview of the derivation of a generalised transition function in Section 2. Two specific transition functions are then considered. In Section 3 the numerical scheme used for computations is derived. Section 4 sees the age structured model with different transition functions compared with experimental data. The experimental data concerns a batch experiment which was conducted using a mouse-mouse hybridoma cell line (mm321) [25]. The findings of this paper are then summarised together with ideas for future work.

## Model Outline

### 1 Age structured model

**Figure 1.Overview:**
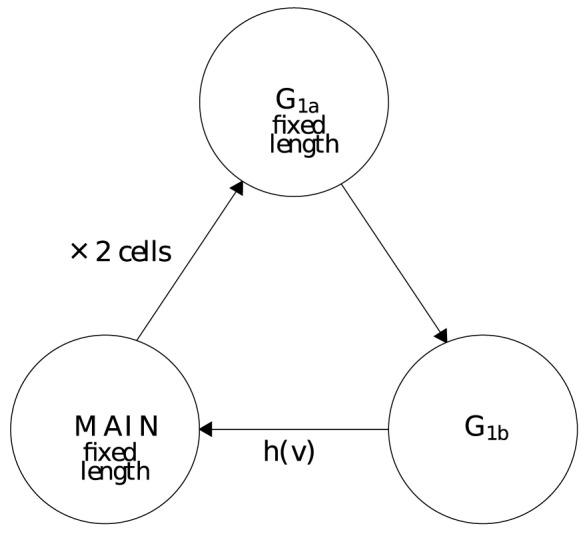
of a three compartment age structured model.

The model considered in this paper is divided into three, age-structured sections, 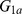, 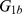 and 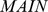 as depicted in Figure 1. The 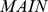 compartment contains cells in the 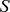, 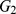 and 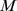 phases of the cell cycle, it is at the end of this compartment cell division occurs.

The 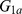 section contains cells which have just undergone division. Cells that are in 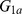 are not able to progress further round the cell cycle until a fixed time period has elapsed, this represents the minimum age a cell can start replicating its DNA. This is biologically realistic as new cells are normally unable to immediately start replicating their DNA. Once cells have progressed to 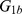 they undergo transition to the 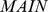 compartment at a rate 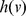, which is often a function of how long the cell has spent in 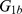. It may also be a function of other factors which effect a cell's progression round the cell cycle such as nutrient levels, the presence of certain drugs, temperature etc. The 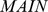 compartment is of fixed duration and can be thought of as merely a time delay from when a cell leaves 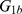 until cell division and entry of the new daughter cells into 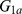. All compartments within this model are of a limited duration, the 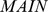 and 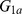 compartments are of a fixed duration and the duration of 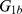 varies from zero to some maximum value, 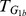. Biologically, any cells remaining in 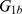 at the end of 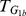 would either die or enter a quiescent phase. Cells in a quiescent phase may be able to rejoin the cycle at a later time. Neither of these scenarios is modelled here.

In this model the non dimensionalised equations governing the population density of cells 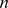 in each phase are given by
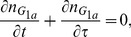
(1)

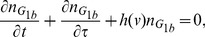
(2)

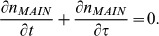
(3)With the corresponding boundary conditions
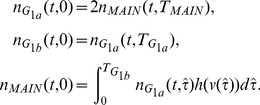
(4)To complete the model the cell distribution at time 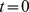 needs to be specified, as we are concerned with the system once it has reached exponential growth and steady ‘phase’ distribution this condition is not important, however for completeness it may be assumed there is a uniform feed of cells into the start of the cell cycle for the first 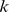 hours,
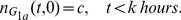
(5)


This model is of a similar structure to most population balance age-structured models such as those presented in [18] and [20] amongst others. In [20] the 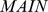 phase is split into three parts 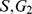 and 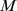, but since our focus is on the total cell population and the fraction of cells in 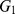, this difference has no impact. A further difference is in the way that [20] model the transition from 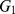, and this will be discussed in greater detail below. In [18], in addition, the 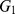 phase is modelled as a single compartment rather than divided into two, 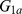 and 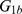.

### 2 G_1_-S Transition functions

**Figure 2:**
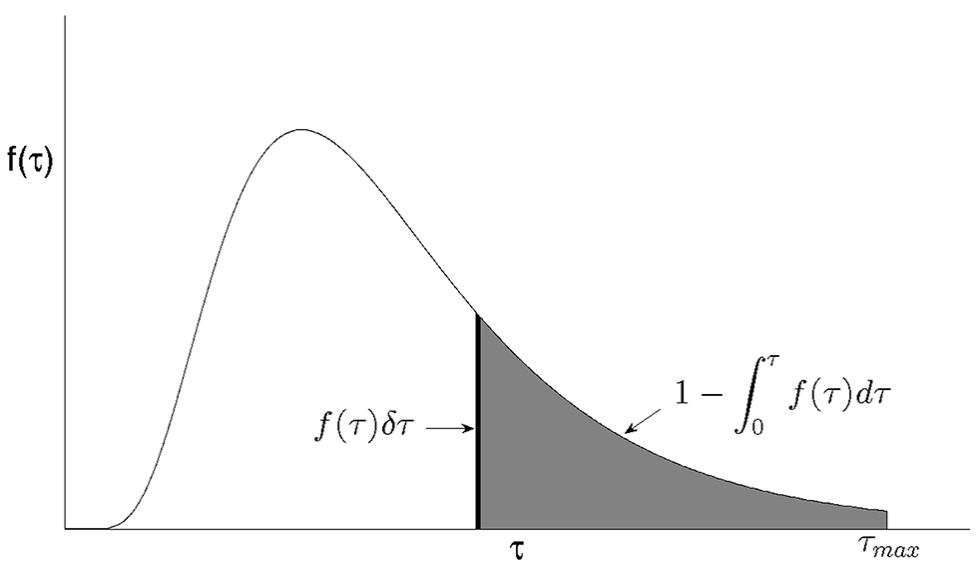
Probability distribution of transition, 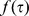 showing the probability that a cell of age 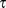 has not yet transitioned (shaded region) and the probability a cell of age 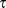 will transition in the time interval 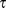 to 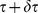 (dark region).

The probability of a cell leaving the 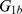 phase and entering the 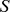 phase via the transition rule is given by some probability distribution function 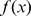 where 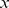 is the variable that determines how likely cells are to undergo transition. Figure 2 gives a graphical representation of such a probability distribution function with phase age 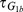 acting as the variable controlling the transition probability. Note that phase age is the length of time a cell spends in a particular phase, For the rest of this paper the subscripts have been removed from the age variable for ease and only used in the case of any ambiguity as to the phase referenced.

If 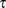 varies by a small amount, 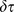, then the probability of cells whose age is between 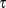 and 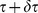 transitioning can be approximated by 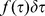. Assuming all cells are capable of transitioning given enough nutrients, the total area under the probability distribution curve is one. Therefore the probability that a cell of age 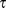 has not yet transitioned is given by 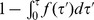. So the fraction of cells, who have not gone through transition, who go through transition when their age changes from 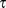 to 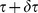 is given by
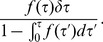
(6)Another way of considering the number of cells going through transition is via a transition rate 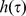. If the fraction of cells who leave in the time period 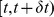 is given by 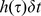, then by definition this must be equal to equation (6). Therefore, in the limit 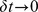,
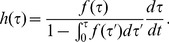
(7)since a cell ages at the same rate as time passes 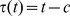 where 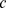 is a constant therefore 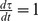 hence equation (7) simplifies to
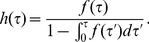
(8)If the cumulative probability of cells transitioning, 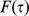, is considered then equation (8) may be expressed as
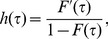
(9)where the dash notation denotes the derivative with respect to 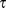. It is this form of the transition rate which will be used herein.

#### 2.1 Specific transition rules

In this paper, we consider two different transition functions, the first assumes that the transition rate is constant, 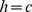, and is therefore independent of the time spent in the 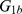 phase. Note the transition rate 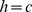 corresponds to a cumulative probability of transition given by 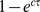. This is the same form of transition discussed in [18]. This transition rule is not biologically realistic as it implies all cells in 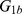 have an equal probability of progressing to the 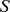 phase regardless of how long they have spent acquiring nutrients and preparing for DNA synthesis.

The second form of transition function that we consider is a sigmoidal transition function. This seems biologically reasonable since this implies that the probability of cells progressing to the 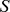 phase immediately after entering 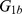 is low due to the limited amount of nutrients they have absorbed. Once the mass of nutrients absorbed reaches some critical value then the probability of transition is likely to increase considerably, however there will always be a few cells which do not progress to the 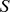 phase regardless of nutrient uptake, thus the sigmoidal function attains a maximum value just under one. It should be noted that a sigmoidal cumulative probability function is in keeping with the phase transition seen in cell populations which have been modelled using the kinetics and chemical processes within the cell [26] and [27]. Here we propose a new sigmoidal transition rule governing the probability of transition is proposed, which unlike the one considered in [24] may be non-dimensionalised so there is only one independent parameter, reducing the number of parameters that need to be fitted.

Since a very small proportion of cells of 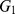 phase age zero it is reasonable to expect that the cumulative distribution function should be non-zero at 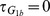. Furthermore, as discussed earlier, some cells will not transition and enter a quiescent state so the cumulative distribution for 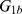 remains less than one for all 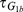. Therefore, the the cumulative distribution function given by
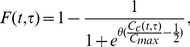
(10)is considered. Here, 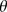 is related to the maximum and minimum values of the cumulative distribution function and 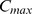 is related to the steepness of the sigmoidal function and 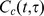 represents the amount of glutamine a cell of age 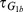 has absorbed at time 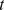. It then follows that, for 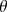 sufficiently large,
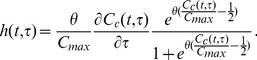
(11)


It is reasonable to assume that the rate of change of glutamine is constant, provided there is a high amount of glutamine available. By making this assumption then 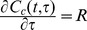 and 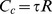 (It is assumed that the cell has not taken absorbed any glutamine prior to entering the 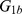 phase, i.e. 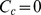 at 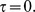). Hence,
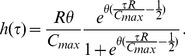
(12)The corresponding non-dimensional form of this equation is given by
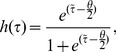
(13)which only has the single parameter 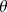 which needs to be fitted. In [28] the following expression for the fraction of cells of age 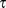 remaining in the 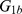 phase, 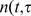), for a given intra cellular glutamine concentration 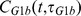 is proposed
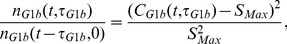
(14)where 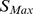 is the maximum glutamine content a cell can have before being forced to go through transition. This leads to the transition function
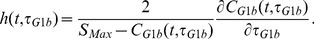
(15)Note 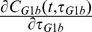 is assumed to always be 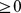 so that the cumulative glutamine never decreases. It can be seen that when 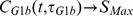, the probability of transition becomes infinite. Despite this singularity at 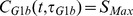 this transition function still provides a very good fit to experimental data [20]. The reasons why this is the case are discussed below.

## Numerical Methods

The system of differential equations governing the simplified system described in Section 1 may be solved analytically for specific initial conditions and short time intervals. However, in order to be able to study and manipulate the model for different transition functions for longer time intervals involving many cell cycles it is necessary to use numerical techniques.

### 3 Derivation of Numerical scheme

In this section a finite difference scheme analogous to the Lax-Wendroff scheme is derived. The Lax-Wendroff scheme was chosen as it is a second order explicit method and as such yields high accuracy for relatively large time steps where there is a rapid change or discontinuity such as the initial flow of cells into the main cycle.

For the 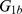 phase equation (2) may be written as
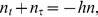
(16)Note for ease the time and age dependence has been omitted together with the phase subscript. Subscripts now denote the partial derivatives. Also 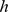 is a function of 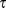 only, furthermore, if the sigmoidal form of the transition rule given in equation (13) is used then
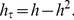
(17)Rearranging and differentiating equation (16) gives
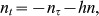
(18a)

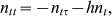
(18b)

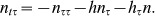
(18c)Which, upon using the Taylor expansion together with (17) yields
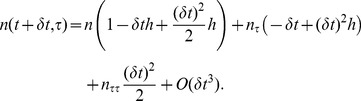
(19)Finally, standard formulae for the first and second derivatives of 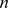 with respect to 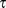 are used, namely
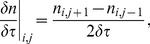
(20)

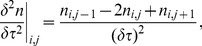
(21)where 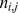 is the cell density of cells aged 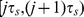 in the time interval 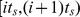 where 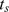 and 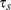 are the length of the discretised elements. This leads to the finite difference scheme
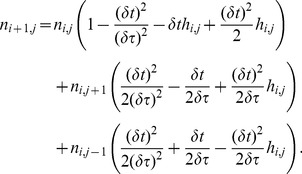
(22)Because of the ‘dispersive’ nature of any numerical difference scheme if 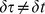 additional errors are introduced at each time step. For example if at 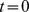 all cells are age zero and the age step is set to 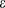 and the time step set to 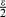, then after evolving the system for one time step there would be cells whose age is 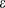, this clearly makes no sense. Similarly if the time step is set to 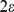 after one step there are no cells present whose age is 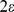 since 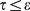 for all cells. Hence, additional interpolation is required if the age and time steps are not equal. By setting 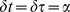 equation(24) becomes
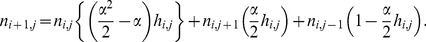
(23)


#### 3.1 Stability of the numerical system

**Figure 3:**
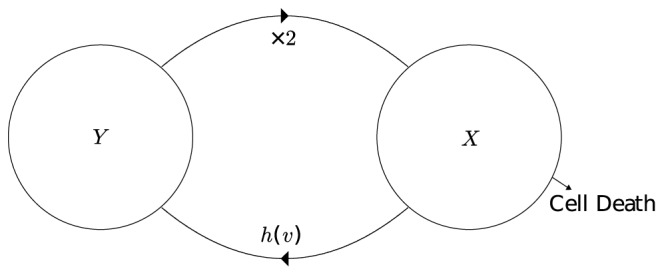
Two Compartment Model.

For a numerical scheme to produce accurate solutions to a partial differential equation, not only must the error at each time step be small enough, any errors must not grow exponentially, i.e. the numerical scheme must also be stable. If the nutrient supply is unlimited and uptake is uniform then the cell cycle may be simplified into two ‘phases’, 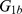 on it's own and the remaining phases all put together. A two compartment model is not suitable for analysing the dynamics of a population of cells as too much information is lost by combining the 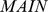 phase and 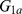 phases of the model discussed in Section 1, in particular the timing of the cell division. However, a two compartment model is sufficient for conducting a stability analysis. Once the system has reached steady growth (i.e. no further input from 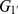) then it may be represented as shown in Figure 3 where 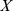 and 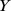 represent the two ‘phases’. To perform the stability analysis the time step matrix is constructed, the norm of which is shown to be bounded. It is helpful to start by defining some notation.

#### Notation

If the numerical scheme is discretised into elements of time of length 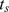 and age elements of length 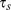 then let cells in phase 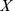 of age 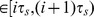 in the time interval 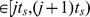 be denoted by 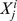. Also let all cells in phase 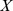 in the time interval 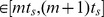 be denoted by 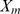, where 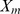 is now a column vector. Also assume the time line is moved such that at 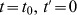, where 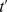 is the time used for the purposes of the subscript; for convenience the 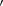 notation is now dropped.

#### Construction of time step matrix

Let the maximum durations of the 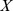 and 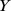 phases be 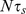 and 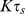 respectively then at time 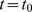,
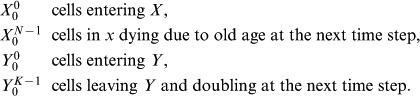
(24)Clearly,
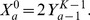
(25)Also the cells entering 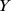 are a function of the cells who were in 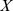 at the previous time step, therefore
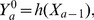
(26)where 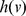 is the probability of transition from 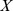 to 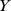. since nothing happens to the cells during their time in 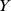, it can be thought of as merely a time delay phase, therefore
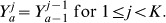
(27)Note, the inequality is strictly less than 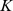 as cells of age 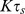 have undergone division and the offspring are now in 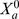.

Assuming a finite central difference scheme is used for calculating the cell densities in the 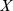 phase then
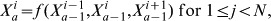
(28)and
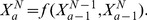
(29)From equations (25) and (27) it is clear that
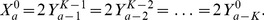
(30)Now using equation (26) yields
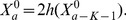
(31)
Equations (25–29) may be expressed in matrix notation as
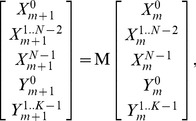
(32)where 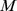 is an 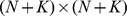 matrix. To prove the numerical scheme is stable it is sufficient to show [29] that the norm of 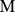 in equation (32) satisfies
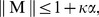
(33)where 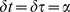 and 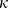 is a constant independent of 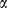. It can be shown that if the trapezium rule is used for approximating equation (26) then the norm of 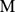 is given by
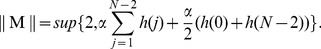
(34)For the transition functions considered 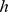 is monotonically increasing so
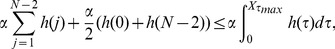
(35)it is therefore sufficient to show 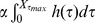 remains bounded. For the sigmoidal transition rule
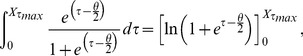
(36)which for typical 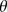 values this is approximately equal to 
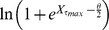
. For 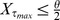 then
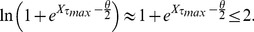
(37)For 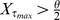 then
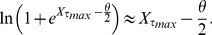
(38)Thus, in all cases 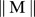 remains bounded. In most cases 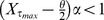, this leads to a stronger constraint on the bound i.e. 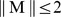.

## Results

In Section 4 it is shown that regardless of whether a constant or a sigmoidal transition rule is used, it is possible to fit the model to a growth curve from experimental data. It is then shown in Section 5 that whilst the different transition functions result in the same growth curve, the fraction of cells in each phase differs.

### 4 Model validation

Experimental data from [25] was chosen and concerns a batch experiment which was conducted using a mouse-mouse hybridoma cell line (mm321). In this experiment 28% of the starting cell population did not divide but remained viable, 36% of the starting population were evenly distributed in the 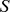 phase of the cell cycle and the remaining 36% were initially at the beginning of the 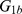 phase. For the purposes of modelling it was assumed the cells starting in the 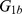 phase were of a phase age between zero and two hours. The numerical scheme described in Section 3, was implemented using both sigmoidal and constant transition rules. Parameters for the length of different phases were taken from [20], and are stated in Table 1. The 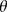 and 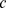 parameters were allowed to vary in the sigmoidal and constant transition rules respectively, until a best fit had been obtained. Several starting values for 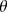 and 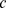 were used in the optimizations of the fits to ensure the global best fits had been found for each transition rule and that the results were not a local minimum. Optimizations were carried out using Matlab's [30] least squares curve fitting algorithm 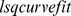. The Matlab code for these optimizations is available from [31].

**Table 1:** Parameters from [20].

Parameter	Notation	Value
Maximum age in 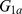 phase	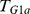	2.5 hours
Maximum age in 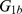 phase	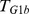	10 hours
Maximum age in 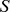 phase	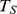	5 hours
Maximum age in 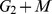 phase	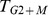	4 hours

**Figure 4:**
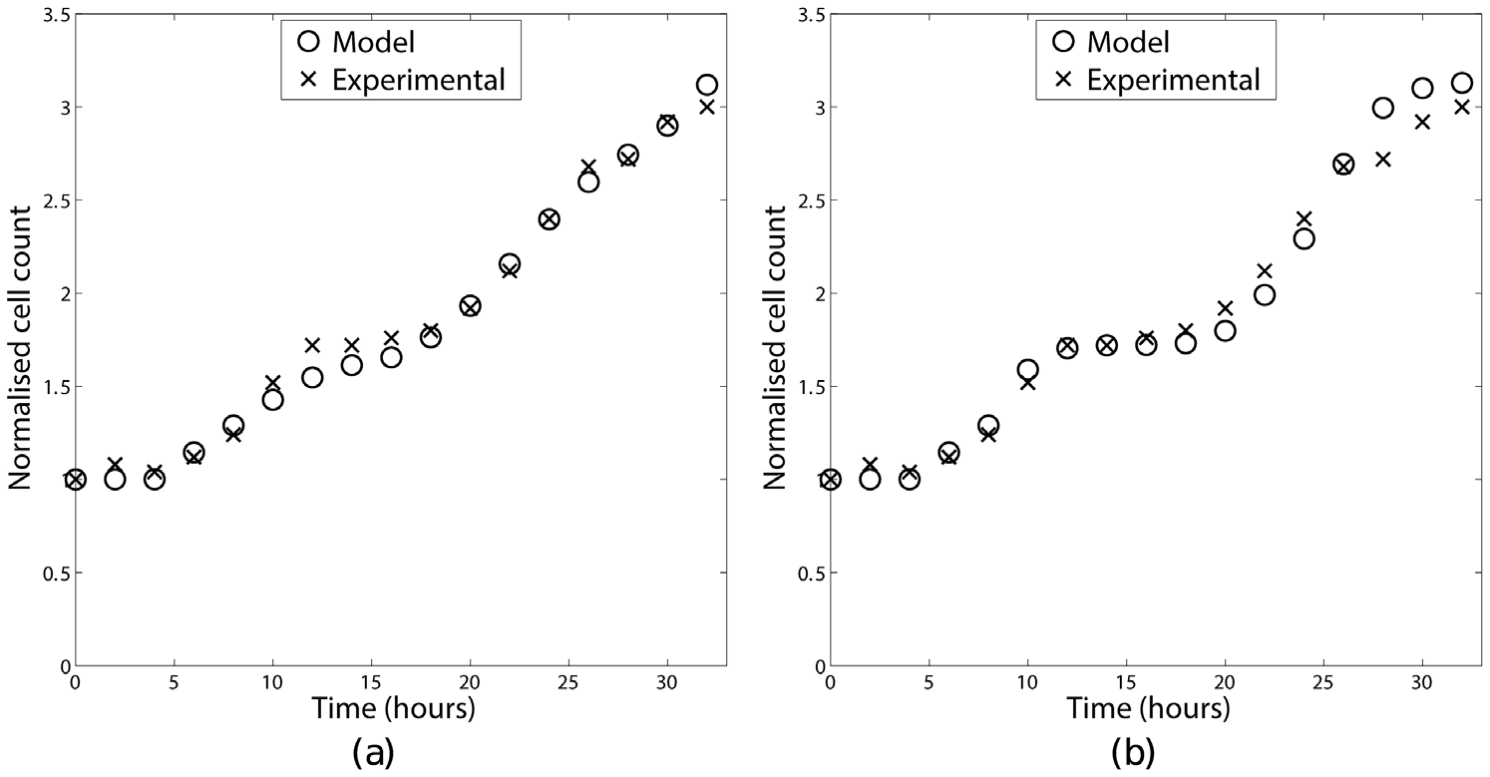
Growth curves produced by using a constant transition rule (a) and a sigmoidal transition rule (b) fitted against experimental batch data presented in [20].

As can be seen in Figure 4, both the constant transition rule (Figure 4a) and the sigmoidal rule (Figure 4b) provide a good fit to the experimental data resulting in residual norm values of 0.1 and 0.2 respectively. The parameters in Table 1 were varied by 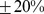. Different values for the Table 1 parameters resulted in different values for the fitted parameters (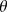 and 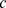) values but did not significantly change the goodness of the fit shown in Figure 4 with no residual norms exceeding 0.2. Note that the model did not impose any restrictions on the available nutrients, indicating nutrients were not a limiting factor for cell growth over the course of the experiment. This suggests, that if population growth is the only concern, that a constant transition rule is sufficient.

### 5 The effect of the transition function

Although the effect of the different transition rules is not apparent in the fitting to the experimental growth curve, here we show that the transition rule does impact on the phase distribution of cells.

In the experimental data used to fit the model the initial population of cells was partially synchronised using a thymidine double block. This partial synchronisation meant the initial population of cells was situated in the 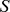 phase and the latter part of the 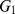 phase, 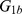. It therefore seems reasonable to assume most cells will initially progress round the cycle in a group this would result in the phase distribution being oscillatory. The oscillations would be expected to decay slowly as the synchronicity of the cell population was lost. Such oscillations may be one cause for apparent ‘errors’ in phase distributions obtained from such experiments as the timing of observations would need to occur at known positions on the oscillation, the period of which may not be known. To fully appreciate the differences these transition functions have on the underlying model properties the percentages of cells in each compartment may be compared once transient oscillations have decayed and the system has reached a steady state of phase distributions. The time scale required for the transient oscillations to have decayed sufficiently is of the order of 500 hours and as such it is not feasible to obtain experimental data.

**Figure 5:**
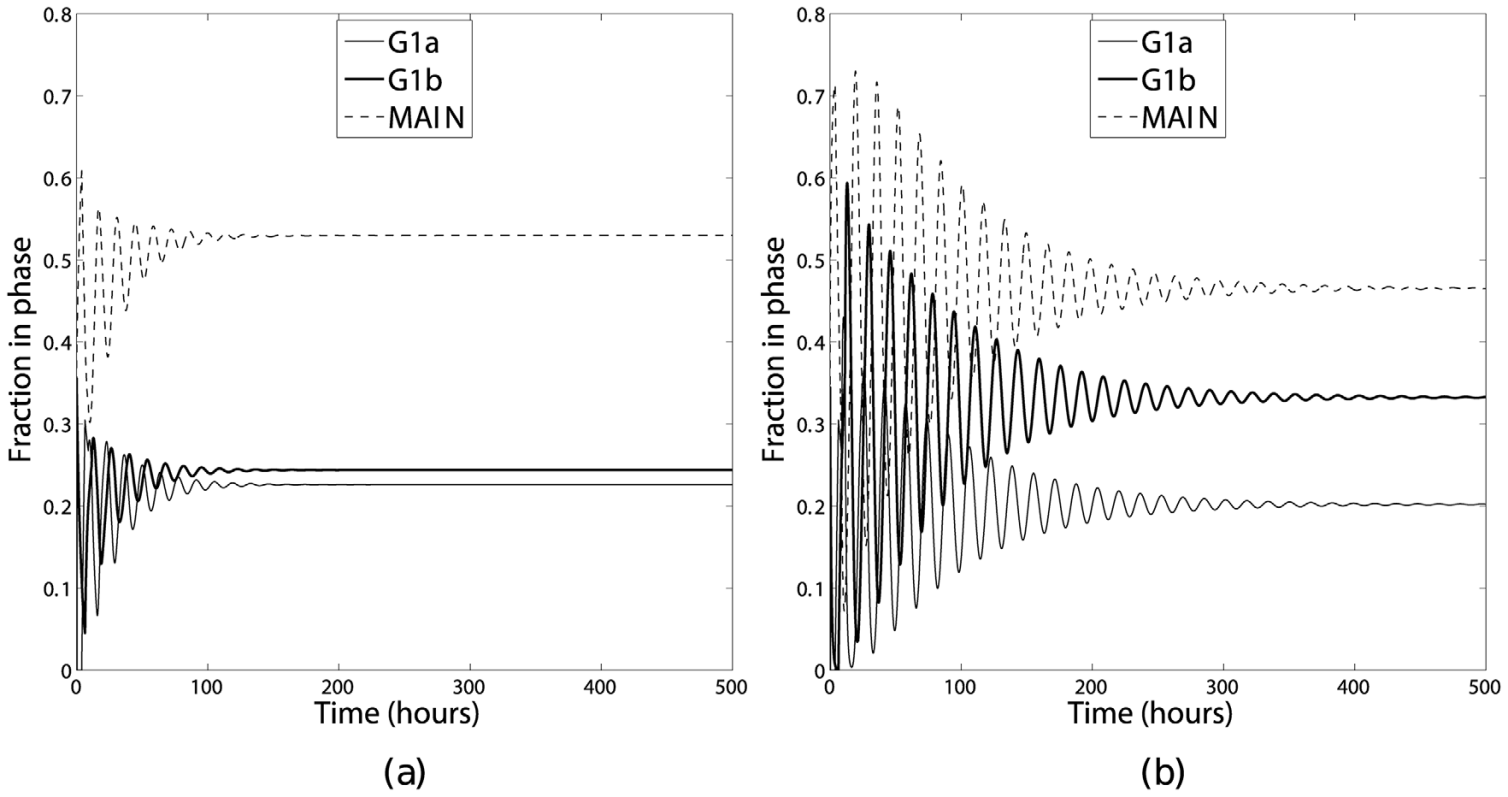
Proportions of cells in each phase using a constant transition rule (a) and a sigmoidal transition rule (b).

**Figure 6:**
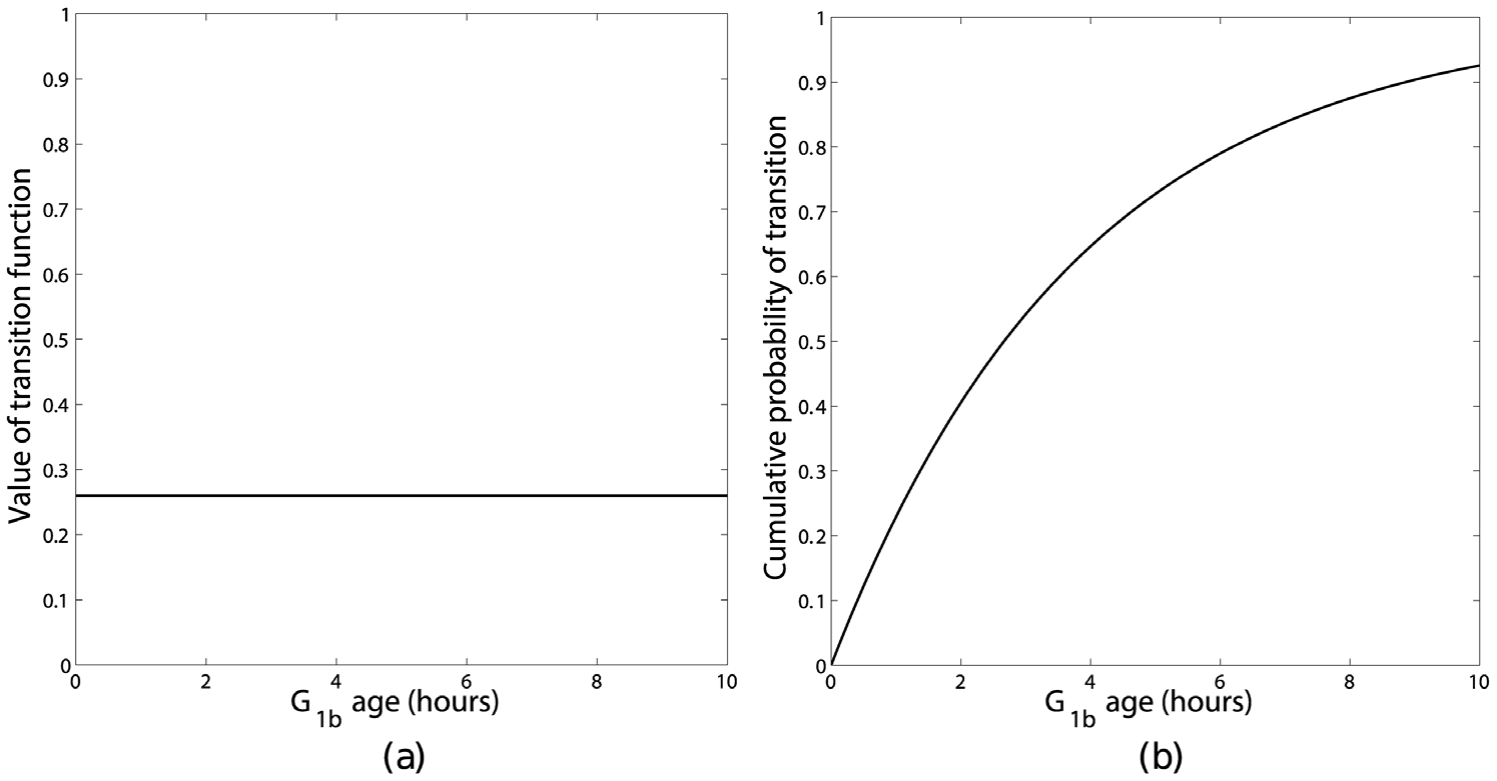
Constant transition function (a) with the corresponding cumulative probability of transition (b) as a function of 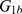 age.

**Figure 7:**
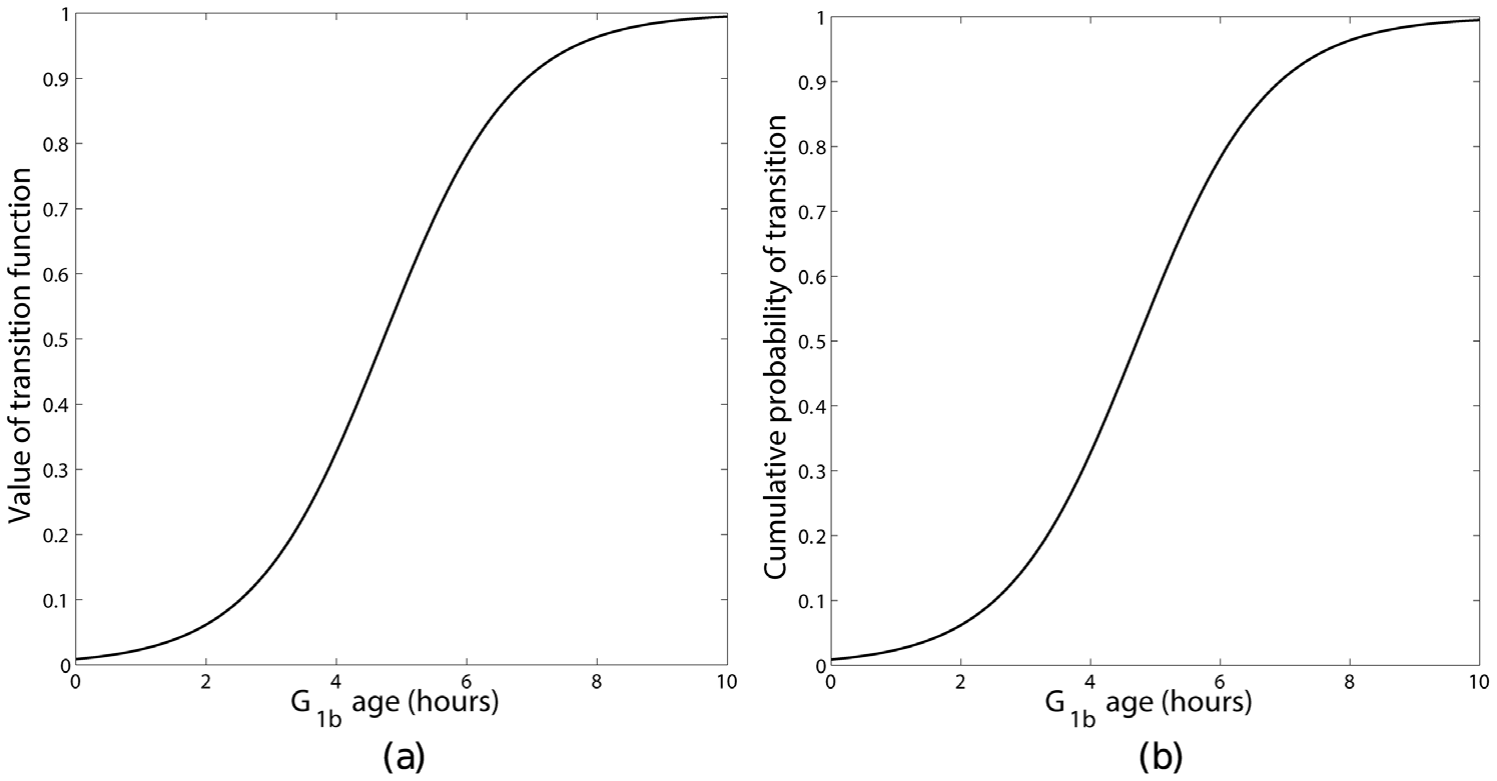
Sigmoidal transition function (a) with the corresponding cumulative probability of transition (b) as a function of 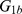 age.

In order to investigate this, the mathematical model was numerically integrated using the same parameters and initial conditions used in Section 4 for long enough that a steady phase distribution had been obtained. The results are shown in Figure 5. These two sets of results differ in two key ways. Firstly, both simulations initially show an oscillation in the phase distribution, however the rate of decay of the oscillations depends on the transition function chosen, with the oscillations decaying much more slowly for a sigmoidal transition function. The difference in the decay rates may be appreciated by considering the area under the cumulative probability function for the different transition functions (Figures 6 and 7). For a steep sigmoidal probability distribution function the area under the curve initially increases slowly then has a rapid increase for a short time interval then returns to a slow increase as shown in Figure 7. This rapid increase would result in the majority of the population remaining in a group as it progressed round the cycle, with each complete cycle dispersing slightly due to the ages corresponding to a low probability of transition. With the value of the constant transition function used in this simulation the area under the corresponding cumulative probability distribution function does not change as rapidly as with the sigmoidal function as shown in Figure 6. This results in the population of cells transitioning more evenly, leading to a more rapid de-synchronisation. Secondly, once the transient oscillations have decayed the percentages of cells in each of the model's ‘phases’ differ: in the sigmoidal transition rule there are 20.2%, 33.3% and 46.5% in the 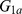, 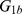 and 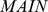 phases respectively, whereas in the constant transition rule these change to 22.6%, 24.4% and 53.0%.

## Discussion

In this paper an age-structured cell cycle model has been considered with particular emphasis on the 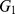-S checkpoint. By considering the probability of cells transitioning at the 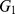-S checkpoint, different transition functions have been obtained. A suitable numerical scheme for the resulting PDEs has been derived and shown to be numerically stable. This numerical scheme has then been used to look at the effects of the different transition functions on the phase distribution of the cell population.

The model shows there is a noticeable change in the proportion of cells in each phase for the two different transition functions considered. The sigmoidal transition function predicts 53.5% of the cell population being in the 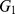 phase, whilst the constant transition function places 47.0% of cells in the 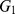 phase.

As mentioned previously the efficacy of chemotherapy treatments and the radiosensitivity of cells varies according to a cell's position in the cell cycle. Since the relationship between cell phase and efficacy may be non-linear a small difference in phase distribution may produce a large change in the efficacy of treatments resulting in the model producing results outside the bounds of experimental error. Therefore, the difference in the phase distributions produced by this model, using the different transition functions, will effect the model's ability to accurately represent the effects of a given treatment on a population of cells. Consequently, it is important to ascertain the correct transition function if such models are to be used to give a quantitative prediction of the cell population's response to treatments.

Improvements in techniques may reduce the level of potential error in phase distributions obtained experimentally, this may allow some transition functions to be discounted.

It may also be possible to rigorously derive the form of the transition function for a population of cells by considering the chemical kinetics of a single cell [26].

Whilst there is no consensus on the error on cell phase distributions obtained using flow cytometry [32] the difference in phase distributions produced by the model with the different transition rules lie within the typical bounds of current experimental error ([33], [34] and [32]). As noted in Section 5 the difficulty of measuring the phase distribution may be compounded by underlying oscillations induced by the blocking. Thus, the form of the probability distribution function controlling the 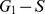 checkpoint in an age structured population balance model has little impact on the models ability to fit to experimental data. The lack of effect of the form of the probability transition function explains why the quadratic transition function used in [20] fitted experimental data despite having a singularity. As such a simplified transition function may be used to gain a qualitative understanding of the dynamics of a population of cells.
